# CaMAPK1 Plays a Vital Role in the Regulation of Resistance to *Ralstonia solanacearum* Infection and Tolerance to Heat Stress

**DOI:** 10.3390/plants13131775

**Published:** 2024-06-27

**Authors:** Lanping Shi, Wei Shi, Zhengkun Qiu, Shuangshuang Yan, Zhiqin Liu, Bihao Cao

**Affiliations:** 1 Key Laboratory of Biology and Genetic Improvement of Horticultural Crops (South China), Ministry of Agriculture and Rural Affairs, College of Horticulture, South China Agricultural University, Guangzhou 510642, China; slpfujian@126.com (L.S.); qiuzhengkun@scau.edu.cn (Z.Q.); ssyan@scau.edu.cn (S.Y.); 2 Key Laboratory of Applied Genetics of Universities in Fujian Province, Fujian Agriculture and Forestry University, Fuzhou 350002, China; wayneshi1101@163.com; 3 College of Agriculture, Fujian Agriculture and Forestry University, Fuzhou 350002, China

**Keywords:** *Capsicum annuum*, MAPK, defense signaling, disease resistance, thermotolerance

## Abstract

As an important member of mitogen-activated protein kinase (MAPK) cascades, MAPKs play an important role in plant defense response against biotic and abiotic stresses; however, the involvement of the majority of the MAPK family members against *Ralstonia solanacearum* and heat stress (HS) remains poorly understood. In the present study, CaMAPK1 was identified from the genome of pepper and its function against *R. solanacearum* and HS was analyzed. The transcript accumulations of *CaMAPK1* and the activities of its native promoter were both significantly induced by *R. solanacearum* inoculation, HS, and the application of exogenous hormones, including SA, MeJA, and ABA. Transient expression of *CaMAPK1* showed that CaMAPK1 can be targeted throughout the whole cells in *Nicotiana benthamiana* and triggered chlorosis and hypersensitive response-like cell death in pepper leaves, accompanied by the accumulation of H_2_O_2_, and the up-regulations of hormones- and H_2_O_2_-associated marker genes. The knock-down of *CaMAPK1* enhanced the susceptibility to *R. solanacearum* partially by down-regulating the expression of hormones- and H_2_O_2_-related genes and impairing the thermotolerance of pepper probably by attenuating *CaHSFA2* and *CaHSP70-1* transcripts. Taken together, our results revealed that CaMAPK1 is regulated by SA, JA, and ABA signaling and coordinates responses to *R. solanacearum* infection and HS in pepper.

## 1. Introduction

As sessile organisms, plants are frequently attacked by various kinds of pathogens with different lifestyles, especially when plants are exposed to unfavorable environments. Once attacked by the invading pathogen, plants usually suffer from serious disease. To survive, plants have developed a sophisticated and efficient defense system, which is considered to be mediated by a two-layered innate immune system, including pathogen-associated microbial patterns (PAMPs)-triggered immunity (PTI) and effector-triggered immunity (ETI) [1,2]. The conserved PAMPs are perceived by specific plant pattern recognition receptors at the cell surface, while effectors are recognized by the intracellular R proteins, which are generally coupled with a hypersensitive response (HR) in the infection site to limit the propagation of pathogens or even kill the pathogens. Recent studies showed that PTI and ETI are inseparably interconnected and can reinforce each other to confer robust plant resistance to disease, although ETI is thought to be more robust, intense, and prolonged than PTI [3,4]. It is well established that the activation of PTI and ETI triggered numerous immune responses, including the rapid influx of Ca^2+^, the activation of mitogen-activated protein kinase (MAPK) cascades, transcriptional reprogramming of defense-associated genes, the burst of reactive oxygen species (ROS), the generation of hypersensitive response-like cell death, and also the activation of defense hormones pathways, including salicylic acid (SA), Jasmonic acid (JA), and abscisic acid (ABA) [4,5,6].

The plant immune system possesses several integrated signaling networks, which are partially regulated by protein kinases. One of the most crucial protein kinase-based amplification cascades is the MAPK cascade [7,8]. The MAPK cascade comprises three protein kinases that activate each other sequentially by phosphorylation: an MAP kinase kinase kinase (MAPKKK) activates an MAP kinase kinase (MAPKK), which then activates an MAPK by phosphorylating tyrosine and threonine residues in the TEY/TDY motif of MAPK [7,8]. An activated MAPK phosphorylates specific substrates, such as transcription factors and enzymes, subsequently triggering cellular responses. It is well established that MAPK cascades play vital roles in regulating defense response against biotic and abiotic stresses, and the roles of MAPK cascades were suggested to be conserved across different plant species [9]. Interestingly, it is considered that the closest algal relatives of land plants were considered to co-express MAPK with environmental signaling responders [10]. *Arabidopsis* MPK4 and MPK6 were found to be induced in transcriptional levels exposed to *Pseudomonas syringae* pv *syringae* and positively regulated the resistance [11]. The complete plant MAP kinase cascade (MEKK1, MKK4/MKK5, and MPK3/MPK6) functions downstream of the flagellin receptor FLS2 and confers resistance to both bacterial and fungal pathogens in *Arabidopsis*. Similarly, soybean GmMPK3 and GmMPK6 positively regulated the immune response of soybean against soybean cyst nematode (SCN) by directly interacting with and phosphorylating GmCDL1, a member of the receptor-like cytoplasmic kinase (RLCK) subfamily VII, and prevented its proteasome-mediated degradation [12]. MPK kinases were also found to function in the maintaining balance between plant growth and defense. *Arabidopsis* MPK3/MPK6 underlines PTI-mediated ETI suppression via forming a module with WRKYs and PP2Cs and is essential for maintaining plant fitness during ETI [13]. Although MAPK activation in response to abiotic stresses is weaker than that activated by PAMPs or pathogen infection, MAPKs also play important roles in plant abiotic stress response as well [14]. MPK3/MPK5 cascade negatively regulates the tolerance of plants to freezing by establishing the ICE1 protein via phosphorylation [15]. In addition, the MEKK1–MKK2–MPK4 cascade can suppress the activity of MPK3/MPK6 to enhance plant cold tolerance [16]. Maize ZmMPK20 enhances plant thermotolerance by negatively regulating high-temperature-induced stomatal opening and balances water loss and leaf temperature [17]. However, the potential roles of the majority of MAPK members in defense remain largely unknown, particularly in non-model plants, including pepper.

Pepper (*Capsicum annuum* L.) is considered one of the most important vegetables worldwide. However, it frequently suffers from several soil-borne pathogens, including *Ralstonia solanacearum*, especially in environments with a high temperature and high humidity. Bacteria from the *R. solanacearum* species complex (RSSC) are soil-borne plant pathogens responsible for bacterial wilt in more than 250 species, including pepper, tomato, and potato [18]. Due to its aggressiveness, widespread geographical distribution, and broad host range, Ralstonia ranks among the most devastating plant pathogenic bacteria [18]. A genome-wide analysis showed that *Arabidopsis* contains 20 putative MAPKs [19], while a total of 19 MAPKs were identified in the pepper genome [20]. However, only a small number of MAPKs in pepper have been identified and functionally analyzed so far. For instance, pepper MAPKs CaMK1 and CaMK2 interacted with CaWRKYa and phosphorylated the SP clusters of CaWRKYa, which is involved in the resistance of pepper plants in response to tobacco mosaic virus (TMV) infection [21]. Additionally, CaMAPK7 was reported to participate in the defense response of pepper against *R. solanacearum* by indirectly modifying the binding of CaWRKY40 to its downstream targets. Furthermore, CaDIMK1 (*Capsicum annuum* drought-induced MAP kinase 1) acts as a positive modulator of drought tolerance and ABA transduction in pepper plants [22]. However, the majority of MAPK kinases have not been characterized in pepper yet, especially their response to *R. solanacearum* infection. In this study, CaMAPK1, a member of the MAPK family in pepper, was isolated and its expression profiles in response to *R. solanacearum* inoculation, heat stress, and exogenously application of hormones were studied. In addition, the roles of CaMAPK1 in the induction of hypersensitive response-like cell death and in response to *R. solanacearum* inoculation and heat stress were also preliminarily analyzed.

## 2. Results

### 2.1. The Sequence Analysis of CaMAPK1

**Figure 1:**
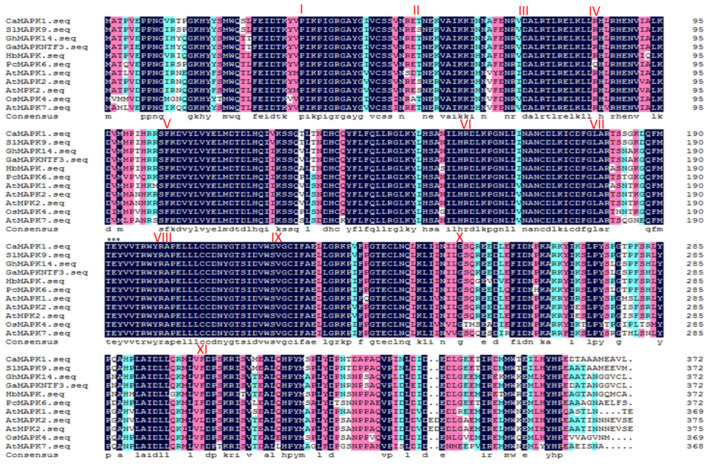
Amino acid sequence arrangements of CaMAPK1 protein and its orthologs from other plant species. Amino acid sequence alignments of CaMAPK1 and its orthologs from tomato (SlMAPK9), cotton (GhMAPK14), Arabidopsis (AtMAPK1 and AtMAPK2), rice (OsMAPK4), and others. The 11 conserved domains (I–XI) present in the serine/threonine protein kinases are denoted by Roman numerals. The conserved threonine and tyrosine residues are indicated by asterisks (***).

Mitogen-activated protein kinases have been reported to play a vital role in the plant immune response against pathogens. However, there is limited knowledge regarding the specific role of MAPKs in plant immunity against *Ralstonia solanacearum*, especially in pepper. To identify MAPK that might function in pepper immunity in response to *R. solanacearum*, we searched the RNA-seq data previously published [23]. CaMAPK1, which exhibited an up-regulated expression pattern in the hypocotyl of pepper plants infected by *R. solanacearum*, aroused our attention. Similar to other MAPKs, CaMAPK1 contains 11 domains that are found to be conversed among the members of serine/threonine protein kinases along with a TEY motif (Figure 1). The predicted protein size and theoretical pI of CaMAPK1 were 42.7 kDa and 6.32, respectively. The similarity of the CaMAPK1 protein sequence and its orthologs from other plant species was compared and analyzed, and the results indicated that CaMAPK1 shares high amino acid identities (>90%) with MAPKs in other plants, including tomato, tobacco, rice, and Arabidopsis, including a 98% match with SlMAPK9 in tomato.

### 2.2. The Expression Profile of CaMAPK1 in Response to R. solanacearum, Heat Stress, and Exogenous Applied Phytohormones

**Figure 2:**
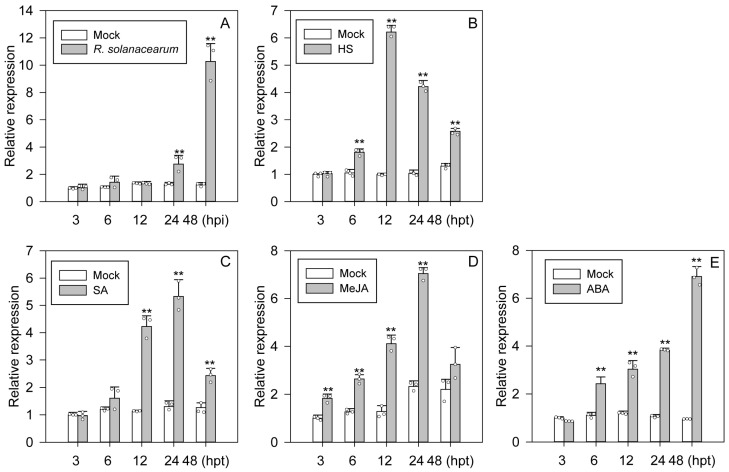
Expression of *CaMAPK1* was up-regulated against pathogen infection, heat stress (HS), and chemical treatments. (**A**) Up-regulation of *CaMAPK1* expression in stems of pepper plants challenged with *Ralstonia solanacearum* in a time course of 48 h. (**B**) Up-regulation of *CaMAPK1* transcript in pepper plants exposed to HS (42 °C). (**C**–**E**) Up-regulation of *CaMAPK1* expression in pepper leaves sprayed with 1 mM SA (**C**), 100 μM MeJA (**D**), and 100 μM ABA (**E**). (**A**–**E**) Hollow dots represent data from 3 independent experiments. *CaMAPK1* transcript levels in stress- or hormone-treated pepper plants were compared with those in mock-treated control plants, which were set to a relative expression level of “1” at 3 h post treatment. All treatments were repeated thrice with similar results. The pepper *CaACTIN* was used as an internal control. Asterisks indicated significant difference as determined by two-tailed *t*-test (** *p* < 0.01).

To further confirm the results of RNA-seqs that *CaMAPK1* transcript was induced against *R. solanacearum* [23], the pepper plants were challenged with *R. solanacearum* by root irrigation, and the abundance of *CaMAPK1* mRNA in the stems was determined at various time intervals post *R. solanacearum* inoculation. The quantitative PCR results showed that *CaMAPK1* expression levels increased significantly from 24 to 48 h post inoculation (Figure 2A). To determine whether CaMAPK1 may participate in the defense response of pepper plants exposed to heat stress (HS), the transcript accumulation of *CaMAPK1* in response to HS was analyzed. Data from quantitative PCR revealed that the *CaMAPK1* transcript began to be significantly induced at 6 h post treatment (hpt) of HS, and the regulation lasted for 48 h and peaked at 12 hpt (Figure 2B). To further assess the possible involvement of CaMAPK1 in signaling pathways utilized by phytohormones, including salicylic acid (SA), Jasmonic acid (JA), and abscisic acid (ABA), the transcript accumulations of *CaMAPK1* in pepper leaves treated with the above-mentioned phytohormones were determined (Figure 2C–E). For SA treatment, the transcript level of *CaMAPK1* was induced at 12 hpt and the induction lasted until 48 hpt (Figure 2C). For MeJA treatments, the *CaMAPK1* transcript began to be up-regulated at 3 h post treatment (hpt), and returned to their ground state both at 48 hpt (Figure 2D). For ABA treatments, *CaMAPK1* transcript began to be upregulated at 6 hpt, the upregulation lasted until 48 h (Figure 2E). The results above suggest that CaMAPK1 might play a role in pepper defense responses against *R. solanacearum* and HS, and also signaling pathways mediated by SA, JA, and ABA.

### 2.3. Analysis of the Promoter Activity of CaMAPK1 against R. solanacearum and Exogenous Hormones

**Figure 3:**
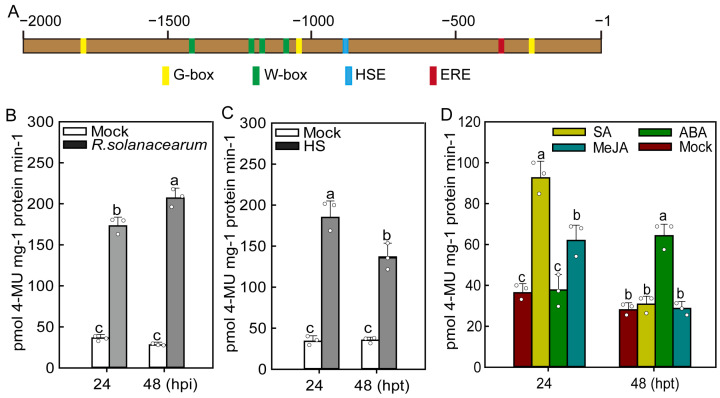
The activities of *CaMAPK1* promoter was enhanced in response to *R. solanacearum* inoculation, heat stress, and chemical treatments. (**A**) The motifs contained in the upstream promoter of *CaMAPK1* with a length of 2000 bp. HSE, heat stress element; ERE, ethylene-responsive element. (**B**,**C**) Enhancements of the activity of *CaMAPK1* promoter at 24 and 48 h post inoculation with *R. solanacearum* (**B**) and exposed to HS (**C**). (**D**) Enhancements of promoter activity of *CaMAPK1* at 24 and 48 h post chemical treatments, including SA, MeJA, and ABA. (**B**–**D**) *Agrobacterium* cells carrying *pCaMAPK1:GUS* were infiltrated into the leaves of pepper plants and the agroinfiltrated plants recovered for 24 h, followed by *R. solanacearum* inoculation, heat stress, and chemical treatments. At 24 and 48 post treatments, the agroinfiltrated leaves were harvested for the measurement of GUS activities. Hollow dots represent data from 3 independent experiments. All treatments were repeated three times with similar results. Different letters indicate significant differences as determined by Fisher’s protected LSD test (*p* < 0.01). LSD, least significant difference.

As the transcriptional expression of a target gene is strictly regulated by the upstream promoter, to investigate the promoter activities of *CaMAPK1* against *R. solanacearum* inoculation and the application of exogenous hormones and confirm the results of expression profiles of *CaMAPK1* we obtained above, the upstream promoter of *CaMAPK1* with a length of 2000 bp was cloned for further study. The bioinformatics analysis indicated that numerous *cis*-acting elements were contained in the *CaMAPK1* promoter, including three G-boxes, four W-boxes, one HSE, and one ERE (Figure 3A), suggesting the expression of *CaMAPK1* might be regulated by different kinds of upstream transcription factors. Next, the full length of the *CaMAPK1* promoter was fused to the reporter gene *GUS* encoding β-glucuronidase to generate a *pCaMAPK1:GUS* reporter construct. An *Agrobacterium*-mediated transient expression system was used to quantify the promoter activity of *CaMAPK1* in response to *R. solanacearum* infection, HS, and the application of exogenous hormones. The results of GUS activities showed that the GUS activities driven by the *CaMAPK1* promoter were significantly induced both at 24 and 48 hpi with *R. solanacearum* or HS, while the increment was enhanced slightly from 24 to 48 h (Figure 3B) for *R. solanacearum*, whereas it weakened for HS (Figure 3C). At 24 h post treatments with hormones, the GUS activities were significantly enhanced in response to SA and MeJA treatments, while no difference was detected for ABA treatment (Figure 3D). However, at 48 h post treatments, the GUS activity was also induced in response to ABA treatments, but not for other hormone treatments, including SA and MeJA (Figure 3D). The results indicated that the alteration of *CaMAPK7* mRNA in response to *R. solanacearum* and the application of exogenous hormones might be regulated by its native promoter.

### 2.4. Transient Expression of CaMAPK1 Induces Cell Death and Defense Responses

**Figure 4:**
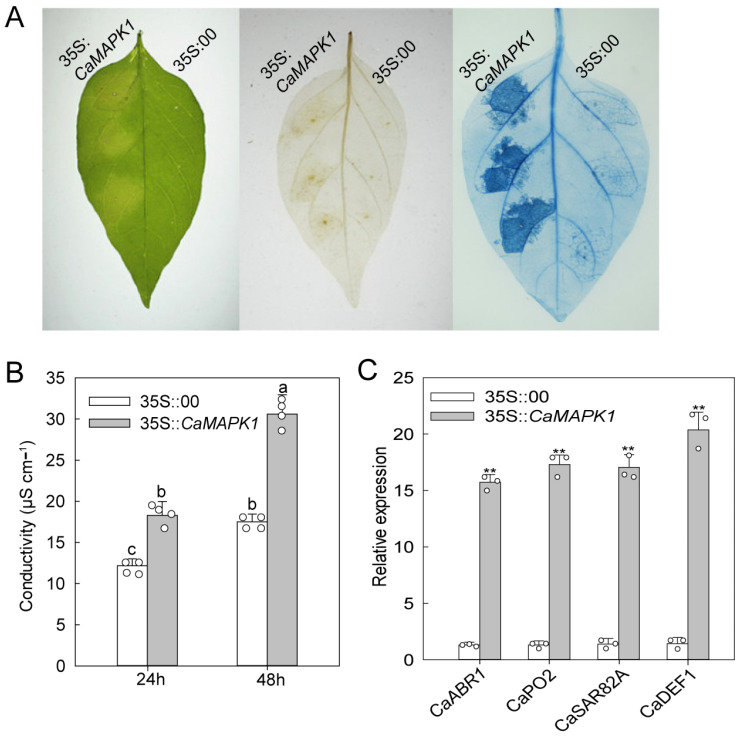
Transient expression of *CaMAPK1* in pepper leaves triggered chlorosis and hypersensitive response-like cell death. (**A**) Pepper leaves transiently transformed with *CaMAPK1* exhibited chlorosis, slight HR-like cell death, and H_2_O_2_ accumulation, confirmed by DAB and trypan blue staining. *CaMAPK1* was transiently expressed in pepper leaves via an *Agrobacterium*-mediated transient expression assay, and DAB and trypan blue staining were performed to evaluate H_2_O_2_ accumulation and cell death, respectively. (**B**) Electrolyte leakage of pepper leaves transiently expressing *CaMAPK1* and empty vector. (**C**) Hormones- and H_2_O_2_-related marker genes were up-regulated by the transient expression of *CaMAPK1* in pepper leaves. The transcript levels of the tested marker genes in pepper leaves transiently expressing empty vector (35S:00) were used as reference, and were set to a relative expression of “1”. (**A**–**C**) Hollow dots represent data from 3 independent experiments. The experiments were repeated three times with similar results. Asterisks indicate significant difference as determined by two-tailed *t*-test (** *p* < 0.01), and different letters indicate significant differences as analyzed using Fisher’s protected LSD test (*p* < 0.05).

To determine the potential role of CaMAPK1 in cell death and defense response, transient expression of *CaMAPK1* was performed in the leaves of pepper plants. Leaves transiently expressing *CaMAPK1* for 48 h triggered an intensive chlorosis and cell death response (Figure 4A). By contrast, pepper leaves transiently expressed with empty vector exhibited no chlorosis and cell death. Cell death was measured by electrolyte leakage from leaf discs agroinfiltrated with *CaMAPK1* or empty vector (Figure 4B). In addition, *Agrobacterium*-mediated transient expression of *CaMAPK1* in pepper leaves induced H_2_O_2_ accumulation at the infiltrated site, as determined by diaminobenzidine (DAB) staining (Figure 4A). H_2_O_2_ from oxidative bursts is known to drive programmed cell death at challenged sites [24]. To determine whether the transient expression of *CaMAPK1* triggered a defense response in pepper leaves, the transcript accumulations of defense-associated genes in pepper leaves transiently transformed with *CaMAPK1* were evaluated. The results show that *CaMAPK1* overexpression in pepper leaves upregulated the expression of defense- and hormones-associated genes, including *CaABR1*, *CaPO2*, *CaSAR82A*, and *CaDEF1* (Figure 4C), suggesting that *CaMAPK1* expression is associated with defense response in pepper leaves.

### 2.5. Knock-Down of CaMAPK1 Attenuated the Resistance of Pepper against R. solanacearum Infection

**Figure 5:**
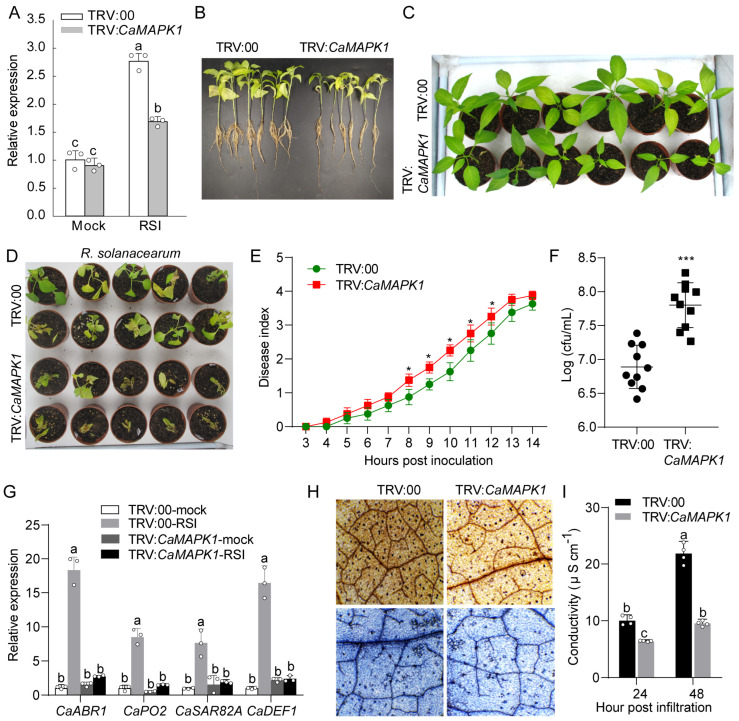
The growth of pepper plant and its disease resistance to *R. solanacearum* were inhibited by *CaMAPK1* silencing. (**A**) The up-regulation of *CaMAPK1* in pepper plants against *R. solanacearum* was significantly inhibited in *CaMAPK1*-silenced pepper plant (TRV:*CaMAPK1*). (**B**,**C**) *CaMAPK1* silencing inhibited the growth of pepper plants, including root (**B**) and leaves (**C**). (**D**) Level of resistance to *R. solanacearum* inoculation determined in *CaMAPK1*-silenced and unsilenced pepper plants at 10 dpi. (**E**,**F**) Progression of bacterial wilt (**E**) and the growth of *R. solanacearum* (**F**) in pepper plants silenced with *CaMAPK1* and empty vector (TRV:00). The tested pepper plants were inoculated with *R. solanacearum* via root irrigation. The disease index was measured from 3 to 14 dpi, and *R. solanacearum* growth was detected at 2 dpi. (**G**) The transcript accumulations of hormones- and H_2_O_2_-related marker genes detected in stems of pepper plants silencing with *CaMAPK1* and empty vector at 2 dpi. (**H**) Decreased H_2_O_2_ production and cell death in leaves of *CaMAPK1*-silenced pepper plants compared with unsilenced plant leaves challenged with *R. solanacearum*. *R. solanacearum*-infected leaves were harvested for DAB and trypan blue staining at 48 hpi, respectively. (**I**) Increment of electrolyte leakage in leaves of pepper plants inoculated with *R. solanacearum* was suppressed by *CaMAPK1*-silencing. (**A**,**G**,**I**) Hollow dots represent data from 3 or 4 independent experiments. (**A**,**E**–**G**,**I**) * *p* < 0.05, *** *p* < 0.001, (two-tailed *t*-test), and different letters indicate significant differences as analyzed using Fisher’s protected LSD test (*p* < 0.05). All the experiments were repeated at least three times with similar results.

To investigate the role of CaMAPK1 in pepper immunity in response to *R. solanacearum* inoculation, a virus-induced gene silencing (VIGS) assay was performed to knock down *CaMAPK1,* and the effect of *CaMAPK1* silencing on pepper immunity against *R. solanacearum* was studied. To this end, a specific fragment of *CaMAPK1* was cloned into the TRV-base vector to generate TRV2:*CaMAPK1*, and together with TRV2:00 or TRV1 vectors, it was used for the VIGS assay. To detect the silencing efficiency of *CaMAPK1* in the VIGS plant, the leaves of generated *CaMAPK1*-silenced plants and unsilenced pepper plants were inoculated with *R. solanacearum* by vein irrigation, and the leaves were harvested for quantitative PCR. The result of the quantitative PCR assay showed that *CaMAPK1* transcript levels in *CaMAPK1*-silenced pepper plants were slight but significantly lower than those in the unsilenced pepper plants. However, the transcript level of *CaMAPK1* in the unsilenced pepper plants was significantly induced upon *R. solanacearum* inoculation, whereas the increment was abolished by *CaMAPK1* silencing (Figure 5A), suggesting the success of gene silencing. We first investigated whether *CaMAPK1* silencing affects the growth and development of pepper plants, including the growths of roots, stems, and leaves. The results showed that *CaMAPK1*-silenced pepper plants exhibited a dwarf phenotype with fewer lateral roots and leaves than unsilenced plants (Figure 5B,C), suggesting that CaMAPK1 may participate in the growth of pepper plants. *R. solanacearum* FJC100301, a highly virulent strain was used to infect VIGS pepper plants to determine whether the knockdown of *CaMAPK1* affects the resistance of pepper plants against *R. solanacearum*. Upon being challenged with *R. solanacearum*, pepper plants silenced with *CaMAPK1* displayed more serious bacterial wilt symptoms than the unsilenced pepper plants at 8 days post inoculation (dpi) (Figure 5D). Simultaneously, higher disease indices and an increment of *R. solanacearum* growth were detected in *CaMAPK1*-silenced pepper plants compared the unsilenced plants are shown in Figure 5E,F. Furthermore, quantitative PCR was performed to detect whether the silencing of *CaMAPK1* alters the expression of defense- and hormones-associated genes during *R. solanacearum* infection, and the results demonstrated that *R. solanacearum* infection significantly induced the expression of *CaABR1*, *CaPO2*, *CaSAR82A*, and *CaDEF1*, and the increments were all significantly suppressed by the silencing of *CaMAPK1* (Figure 5G).

We next examined local defense responses by 3,3-DAB staining of H_2_O_2_ accumulation (dark brown) and HR-like cell death confirmed by trypan blue staining (dark blue) of the leaves infected by *R. solanacearum* (Figure 5H). Since it is reported that plant exhibits similar phenotypes between root inoculation and leaf infiltration [25], the plant leaves were infiltrated with the bacterial suspension using a needleless syringe, and the inoculated leaves were harvested for DAB and trypan blue staining to evaluate the local defense response. Compared with the unsilenced pepper plants, the leaves of *CaMAPK1*-silenced plants infected by *R. solanacearum* exhibit more intense colors of dark brown and blue after DAB and trypan blue staining, respectively (Figure 5H). In addition, lower electrolyte leakage was detected in *CaMAPK1*-silenced pepper plants, compared with that in unsilenced pepper plants (Figure 5I). Taken together, the silencing of *CaMAPK1* in pepper plants seems to decrease the basal defense against *R. solanacearum*, resulting in the inhibition of H_2_O_2_ accumulation, and seems to decrease the ability of plants to respond to *R. solanacearum* recognition by suppressing HR-like cell death.

### 2.6. Silencing of CaMAPK1 Attenuated Thermotolerance of Pepper Plants

**Figure 6:**
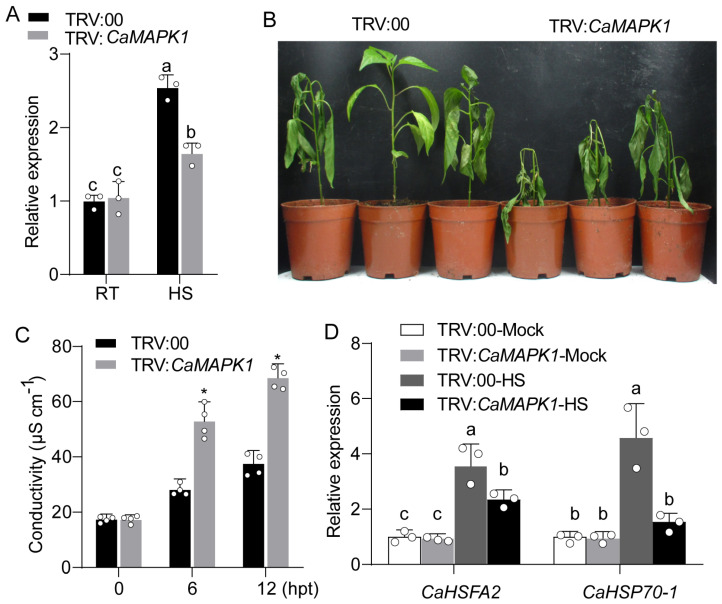
Silencing of *CaMAPK1* impaired pepper thermotolerance. (**A**) Transcript accumulations of *CaMAPK1* in *CaMAPK1*-silenced and unsilenced pepper plants exposed to HS. (**B**) Heat-stress-sensitive phenotype of *CaMAPK1*-silenced pepper plants. *CaMAPK1*-silenced and unsilenced pepper plants were subjected to HS and maintained at 42 °C for 48 h. (**C**) Electrolyte leakage from leaves of *CaMAPK1*-silenced and unsilenced pepper plants exposed at 6 and 12 h post HS treatment. (**D**) Quantitative RT-PCR analysis of heat-stress-related marker genes in HS-treated *CaMAPK1*-silenced and unsilenced pepper plants at 24 hpi. (**A**,**C**,**D**) Hollow dots represent data from 3 or 4 independent experiments. * *p* < 0.05 (two-tailed *t*-test), and different letters indicate significant differences as analyzed using Fisher’s protected LSD test (*p* < 0.05). Experiments in (**A**–**D**) were repeated at least thrice with similar results.

The up-regulation of *CaMAPK1* exposed to HS suggested a potential role of this kinase in thermotolerance. To this end, the VIGS plants were used to study the effect of *CaMAPK1* silencing on pepper thermotolerance. The silencing efficiency of *CaMAPK1* was assayed in VIGS plants upon heat stress treatment by quantitative PCR, and the result revealed that *CaMAPK1* was significantly up-regulated when exposed to HS (42 °C) in unsilenced pepper plants, and the increment was significantly abolished in *CaMAPK1*-silenced pepper plants (Figure 6A), suggesting the success of gene silencing in the tested pepper plants. After HS treatment, the *CaMAPK1*-silenced pepper plants exhibited a more severely injured phenotype with most leaves being withered at 48 hpt (Figure 6B). In addition, compared to the leaves of the unsilenced pepper plants, a high level of electrolyte leakage, indicated by conductivity, was found at 6 and 12 hpt in the leaves of *CaMAPK1*-silenced pepper plants (Figure 6C). Furthermore, quantitative PCR was performed to detect the effect of *CaMAPK1* silencing on the expression of HS-associated genes, including *CaHSFA2* and *CaHSP70-1* [26]. The results indicated that the expression levels of the tested genes in unsilenced pepper plants were significantly induced after heat stress treatment; however, the increments were significantly repressed by the silencing of *CaMAPK1* (Figure 6D). Taken together, these results revealed that CaMAPK1 acts positively in pepper thermotolerance.

## 3. Discussion

### 3.1. CaMAPK1 Acts as a Positive Regulator of Defense Response and Cell Death in Pepper Plants

As the extreme downstream member of MAPKs cascade, MAPKs were reported to function in plant growth, development, and defense responses to stresses, including biotic and abiotic stresses via the phosphorylation of a target substrate. To date, most of the studies of MAPKs have focused on the model plants, including *Arabidopsis* and rice. Identifying the novel MAPK members and exploring their functions in non-model plants such as pepper will benefit from the dissection of MAPK cascade in different plant species. Our previous study found that 19 MAPKs are identified in the genome of pepper; however, only 3 MAPKs out of 19, CaMAPK7, MK1, and MK2 (designated CaMPK3 and CaMPK6-1 in our previous study), were cloned and functionally analyzed. MK1 and MK2 were induced at the transcript level and played roles in response to wounding, UV-C and cold [27]. CaMAPK7 positively regulated the defense response of pepper plants against *R. solanacearum* by indirectly modifying the binding of transcription factor CaWRKY40 to its downstream targets. In the present study, a novel MAPK CaMAPK1 was identified (Figure 1) and its functions in response to *R. solanacearum* and heat stress (42 °C) were studied. MAPKs function in response to stresses and exhibit inducible expression patterns against the corresponding stress in general. Herein, our quantitative PCR results showed that the transcript level of *CaMAPK1* in pepper stem was significantly upregulated against *R. solanacearum* inoculation by root irrigation (Figure 2). A previous study by Du et al. [23] showed that *CaMAPK1* mRNA abundance was significantly induced in the hypocotyl of pepper plants challenged with *R. solanacearum* at the root. However, our previous study found that the *CaMAPK1* transcript level in pepper leaves was slightly induced when the leaves were infected by *R. solanacearum* [20]. The results indicate that the *CaMAPK1* transcript was induced against *R. solanacearum,* and the induction seems to be tissue-associated. Gene transcript expression was strictly modified by the upstream native promoter, and the motifs contained in the promoter were associated with the expression pattern and function of the target gene. Herein, PlantCARE databse (http://bioinformatics.psb.ugent.be/webtools/plantcare/html/) was used to screen the motifs in the *CaMAPK1* promoter and numerous motifs, including transcription factor WRKY binding motif W-box [28], G-box [29], heat shock element HSE [30], and ethylene-responsive ERE box [31], were found. These motifs were reported to play an important role in plant immune response and signaling pathways. Promoter activities analysis of *CaMAPK1* showed that *R. solanacearum* infection, heat stress, and the application of exogenous hormones, SA, MeJA, and ABA, significantly triggered the activities of *CaMAPK1* promoter (Figure 3). *R. solanacearum* infection and transient expression of *CaMAPK1* by agroinfiltration induced intensive HR-like cell death, ROS accumulation, and concomitantly enhanced expression of *CaMAPK1* transcript in pepper leaves (Figure 4). However, cell death and ROS accumulation were dramatically compromised in *CaMAPK1*-silenced pepper leaves, which exhibited an increased susceptibility to *R. solanacearum* infection (Figure 5). Taken together, these results suggest that CaMAPK1 functions as a positive regulator of cell death in pepper plants, and CaMAPK1-dependent cell death may require ROS production at the infection site.

Silencing *CaMAPK1* repressed the resistance of pepper plants against *R. solanacearum*, accompanied with higher growth of *R. solanacearum* and a disease index in *CaMAPK1*-silenced pepper plants, suggesting that CaMAPK1 may positively regulate pepper immunity against *R. solanacearum* via inhibiting the bacterial growth. Interestingly, a study from Kim et al. showed that CaDIMK1 (designed as CaMAPK1 in the present study) functions as a positive regulator of drought stress response and ABA signaling in pepper [22]. As a soil-borne pathogen, *R. solanacearum* invades plants through the root and natural wounds. Once it invades the plant root, *R. solanacearum* propagates in vascular bundles and blocks them via the production of polysaccharides, eventually leading to dehydration-like physiological symptoms. Since CaMAPK1 positively regulated both pepper’s resistance to *R. solanacearum* and its drought stress response, we speculate that CaMAPK1 may contribute to the resistance of pepper plants against bacterial wilt by coupling enhanced immunity and increased dehydration tolerance. Similarly, our previous study indicated that the CaPti1/CaERF3 module positively functions in pepper resistance to bacterial wilt by coupling immunity and dehydration tolerance [32]. However, by which mechanism CaMAPK1 couples immunity and dehydration tolerance needs to be further unraveled.

### 3.2. CaMAPK1 Participates in the Regulation of Thermotolerance of Pepper Plants

Our present data revealed that the transcripts of *CaMAPK1* were up-regulated in pepper plants exposed to heat stress (42 °C) and the increments lasted from 6 h to 48 h (Figure 2). Moreover, silencing *CaMAPK1* impaired the thermotolerance of pepper plants (Figure 6), suggesting that CaMAPK1 plays a positive role in thermotolerance besides confer disease resistance to *R. solanacearum*. Thermotolerance can be induced via transcriptional reprogramming mediated by the heat-shock transcription factors (HSFs) binding to the heat-shock elements in the promoter of *HSP* genes. We found that heat stress up-regulated the transcriptional level of *CaHSFA2* [33], and the up-regulation was significantly impaired by the silencing of *CaMAPK1*, suggesting that the transcriptional up-regulation of CaMAPK1 under heat stress activated the thermotolerance at least partially by modulating the transcript accumulation of *CaHSF2A*. Under HS, heat shock proteins (HSPs) were rapidly synthesized and mitigated the effects of HS on plants and were considered to be responsible for the acquisition of thermotolerance [34]. The present study indicated that the up-regulation of *CaHSP70-1* induced by HS treatment was almost fully suppressed by *CaMAPK1* silencing, suggesting that the expression of *CaHSP70-1* upon heat stress is partially regulated by CaMAPK1. Thus, the data in the present study suggested that decreased thermotolerance by *CaMAPK1* silencing in pepper plants is attributed to the transcriptional regulation of heat tolerance-associated genes, including *CaHSF2A* and *CaHSP70-1*.

### 3.3. CaMAPK1 May Contribute to the Immunity against R. solanacearum Mediated by SA, JA, and ABA

The production of several phytohormones, including SA, JA, and ABA, is frequently induced by the invasion of pathogens. The outputs of hormone signaling are proven to activate the expressions of serials of *PR* genes, leading to the enhanced resistance of plants against pathogens with different lifestyles. In the present study, we found that *CaMAPK1* transcripts and the activities of its native promoter were significantly induced upon *R. solanacearum* inoculation and the application of exogenous hormones, including SA, JA, and ABA (Figure 2 and Figure 3). In addition, overexpression of *CaMAPK1* in pepper leaves triggers the transcript accumulation of hormone-related genes (Figure 4), including ABA-related *CaABR1* [35], SA-associated *CaSAR82A* [35], and JA-related *CaDEF1* [36]. Of note, *R. solanacearum* infection in pepper leaves upregulated the expression of the tested marker genes above, whereas the increments were significantly inhibited in *CaMAPK1*-silenced pepper plants (Figure 5). Taken together, we hypothesize that SA, JA, and ABA regulate the expression of *CaMAPK1*, leading to the activation of downstream defense.

## 4. Materials and Methods

### 4.1. Plant Materials and Pathogen Inoculation

The seeds of pepper cultivated variety HN42 were provided by Fujian Agriculture and Forestry University. Pepper plants were grown in a greenhouse or chambers as previously described [37,38]. *Ralstonia solanacearum* inoculation (soil-drenching inoculation) was performed as previously described [32]. Briefly, the cultured *R. solanacearum* cells (FJC100301) were harvested by centrifugation and suspended in sterilized ddH_2_O (10^8^ colony-forming units per mL, CFU mL^−1^). The roots of pepper plants with fully expanded leaves were damaged in the root by inserting a knife into the soil thrice before *R. solanacearum* inoculation via root irrigation. After inoculation, the infected pepper plants were maintained in the chamber with a temperature of 28 °C and 75% humility. For bacterial quantification, 2.5 μL xylem sap was collected from each plant at 48 h post inoculation (hpi) and used for CFU determination. The disease index was rated daily on a scale ranging from 0 to 4 as previously described [38].

### 4.2. Heat Stress Treatment

Thermotolerance assays were performed as previously described [39]. Briefly, the *CaMAPK1*-silenced and unsilenced pepper plants were kept in the chamber with a temperature of 42 °C for 24 or 48 h, followed by recovery at 28 °C for 2 d.

### 4.3. Quantitative Real-Time PCR

The total RNA of pepper plants was extracted using the reagent TRIZOL then reverse transcribed to complementary DNAs (cDNAs) with MMLV reverse transcriptase. The generated cDNA was diluted 10-fold and used for quantitative PCR. Quantitative PCR was carried out using the commercial SYBR premix according to the instructions of the kits. The mRNA accumulation of *CaACTIN* was used to normalize the relative expression of the tested genes [40]. For each biological replicate, three technical replicates were assayed.

### 4.4. Protein Extraction and Immunoblot Assay

The harvested plant tissues, including stems and leaves, were ground into powder in liquid nitrogen and then homogenized in total protein extraction buffer as previously described. An equal amount of protein for each sample was separated using SDS-PAGE gel and the separated proteins were blotted into the PVDF membranes. The PVDF membranes were probed with the corresponding antibodies at 1:3000 to 1:5000 dilutions.

### 4.5. Subcellular Localization

Subcellular localization assay was performed as in our previous study. Briefly, *Agrobacterium* cells of GV3101 strain harboring *35:CaMAPK7-GFP* were transiently expressed in the leaves of 4–5-week-old N. benthamiana via agroinfiltration. At 36–48 h post transformation, the infiltrated leaves were harvested for fluorescent detection using a confocal microscope (SP8). The emission and excitation wavelengths were 488 and 510–520 nm, respectively.

### 4.6. Agrobacterium-Mediated Transient Expression

*Agrobacterium*-mediated transient expression assay was performed as previously described with slight modification [41,42]. Briefly, *Agrobacterium* cells carrying CaMAPK7-Flag construct were transiently expressed in the fully expanded leaves of pepper plants via agroinfiltration using a needleless syringe and the plants were maintained in the chamber. At 24 h or 48 h post infiltration, the leaves were harvested for further experiments, including histochemical staining, measurement of conductance, and total RNA extraction.

### 4.7. Virus-Induced Gene Silencing in Pepper Plants

The tobacco rattle virus (TRV)-based VIGS system was used to knock down *CaMAPK1* in pepper plants and performed as previously described [43]. The silencing fragment of *CaMAPK1* (the specification of the fragment is confirmed in https://solgenomics.net/tools/blast/, accessed on June 2016) was amplified using the primers listed in Table S1 and cloned into the satellite vector pDONR207. After sequencing to confirm the accuracy of the inserted fragment, *CaMAPK1* silencing fragment was subcloned into pTRV2 vector to generate TRV:*CaMAPK1*. Cells of *Agrobacterium* GV3101 carrying containing pTRV1 and pTRV2:00 or pTRV2:*CaMAPK1* were mixed in 1:1 ratio to a final OD600 of 0.6 and infiltrated into two fully expanded leaves of 2–3-week-old pepper plants. *Agrobacterium*-infiltrated pepper plants were grown at 25 °C with a 16 h light/8 h dark photoperiod cycle and were used after 5–6 weeks of VIGS treatment.

### 4.8. Histochemical Staining

Histochemical staining assays, including DAB and trypan blue staining, were performed as previously described with slight modification [38]. One mg mL^−1^ diaminobenzidine (DAB) or lactophenol-ethanol trypan blue (10 mL of lactic acid, 10 mL of glycerol, 10 g of phenol, 30 mL of absolute ethanol and 10 mg of trypan blue, dissolved in 10 mL of distilled ddH_2_O) was used for DAB and trypan blue staining, respectively. For DAB staining, the stained leaves were cleared by destaining overnight in absolute ethanol. For trypan blue staining, the stained leaves were destained in a chloral hydrate solution (2.5 g of chloral hydrate dissolved in 1 mL of distilled water). The representative phenotypes were photographed with a light microscope (Leica, Wetzlar, Germany).

### 4.9. Statistical Analyses

The differences among multiple groups were indicated by different letters (*p* < 0.01), as calculated with Fisher’s protected least significant difference (LSD) test. The differences between the two groups are indicated by single (statistically significant *p* < 0.05), double (very significant, *p* < 0.01), and three asterisks (extreme significant, *p* < 0.001) (two-tailed *t*-test).

## Data Availability

The raw data supporting the conclusions of this article will be made available by the authors on request.

## Supplementary Materials

The following supporting information can be downloaded at: https://www.mdpi.com/article/10.3390/plants13131775/s1. Table S1. Oligonucleotides used in this study.
